# Antioxidant Effect of the *Castanea sativa* Mill. Leaf Extract on Oxidative Stress Induced upon Human Spermatozoa

**DOI:** 10.1155/2019/8926075

**Published:** 2019-12-19

**Authors:** Marco Biagi, Daria Noto, Maddalena Corsini, Giulia Baini, Daniela Cerretani, Giorgio Cappellucci, Elena Moretti

**Affiliations:** ^1^Department of Physical Sciences, Earth and Environment, University of Siena, Via Laterina 8, Siena 53100, Italy; ^2^Department of Molecular and Developmental Medicine, University of Siena, 53100, Italy; ^3^Department of Biotechnology, Chemistry and Pharmacy, University of Siena, 53100, Italy; ^4^Department of Medical and Surgical Sciences and Neurosciences, University of Siena, Siena 53100, Italy

## Abstract

This study was aimed at evaluating *in vitro* the effects of a 75% *v*/*v* ethanolic extract of leaves of *Castanea sativa* Mill. (var. Bastarda Rossa, Mount Amiata, Tuscany, Italy) on ejaculated human sperm. Total polyphenols and total flavonoids contained in the extract were determined by a colorimetric assay and HPLC-DAD. The DPPH test and electrochemistry were utilized to study the antioxidant activity of the extract. Swim-up-selected sperm from 8 healthy men were treated with the *C. sativa* leaf extract at different dilutions (1 : 100, 1 : 200, and 1 : 500), and sperm motility was assessed following the WHO guidelines. Swim-up-selected spermatozoa were incubated with 100 *μ*M H_2_O_2_ to induce lipid peroxidation (LPO) and with H_2_O_2_ and the leaf extract (1 : 100, 1 : 200, and 1 : 500) to test the antioxidant activity of the extract. The levels of LPO were determined by measuring malondialdehyde (MDA) concentrations. The treated samples were also analyzed by transmission electron microscopy (TEM) for ultrastructural evaluation. The chemical analysis showed that one-third *ca.* of the polyphenols in the *C. sativa* extract were made up of flavonoids, with hyperoside present in high concentration. A good antioxidant activity was demonstrated by both the DPPH test and electrochemical analysis. The *C. sativa* leaf extract did not decrease sperm motility at all tested dilutions. Treatment with H_2_O_2_ alone caused a significant increment in MDA levels (*P* = 0.006993), while the treatment with H_2_O_2_ plus *C. sativa* extract diluted to 1 : 100 and 1 : 200 significantly reduced MDA levels (*P* = 0.01476 and *P* = 0.01571, respectively), with respect to H_2_O_2_ alone. TEM analysis confirmed the protective effect of the extract on damage induced by LPO, in particular that occurring at the plasma membrane level. The *C. sativa* leaf extract could be used in human and farm animal protocols for gamete handling, such as techniques of assisted reproduction and cryopreservation of semen, all conditions in which oxidative stress is exacerbated.

## 1. Introduction

*Castanea sativa* Mill. (*C. sativa*) is a very long-lived tree, belonging to the *Fagaceae* family present in South Europe and Asia. The fruit chestnut is a healthy food and has valuable nutritional qualities. It is noteworthy that when harvesting chestnuts, a great deal of waste products is obtained, such as leaves and inner and outer shells. Therefore, it could be important to try to use also the waste material to find substances that can be utilized in industrial, cosmetic, and nutraceutical fields and other areas, encouraging recycling and a sustainable economy [1].

Extracts of this plant have displayed a clear biological activity; in particular, they have shown cardioprotective [2], antihelminthic, antibacterial, and antiviral effects [3] and neuroprotective activity [4]. Observations recently made by Budriesi et al. [5] in high-fat diet rats suggested that the chestnut extract has the potential to be used in medicine as a dietary supplement in the treatment of obesity complications.

For a long time, chestnut leaf infusions have been used to treat rheumatism, cough, and diarrhea [6]. They also exert calming and sedative action and are an excellent remedy in the treatment of cold and cases of respiratory problems.

Recent studies highlighted the presence of high concentrations of phenolic compounds endowed with antioxidant activity in the chestnut waste material extract [1]. Leaves are rich in tannins (6%-8% of leaf dry weight) including ellagic acid, as well as flavonoids (0.1-0.3%) such as rutin, hesperidin, quercetin, apigenin, morin, galangin, and kaempferol [3, 7]. Cinnamic derivatives, such as chlorogenic acid, are also present [7]. It is known that these compounds are involved in the defense against the oxidative damage and are able to protect the main targets of reactive oxygen species (ROS), which are DNA, proteins, and lipids [8]. The scavenging activity of these compounds is due to the presence of hydroxyl groups [9].

Therefore, *C. sativa* leaf extracts are used in different applications. For example, they are able to protect from oxidative damage the DNA of pancreatic *β*-cells treated *in vitro* with the diabetogenic agent streptozotocin [10]. Quave et al. [11] reported that *C. sativa* leaf extracts rich in pentacyclic triterpenes, such as ursene and oleanane derivatives, blocked the *Staphylococcus aureus* pathogenic activity and virulence. In addition, leaf phenolic extracts of the Italian product Marrone di Roccadaspide (Campania region, Italy) showed a protective effect against UVB-induced damage on human keratinocytes [12].

It is known that oxidative stress results from alterations in the complicated balance between reactive oxygen species (ROS) generation and elimination. The presence of physiological levels of ROS supports some main functions of sperm, such as motility, capacitation, acrosome reaction, zona pellucida binding, and oocyte fusion [13]. However, an uncontrolled ROS production and/or the defects in the balance between ROS concentration and the antioxidant scavenging system affect the normal sperm function damaging sperm membranes, DNA, and proteins [14]. In particular, sperm membranes are rich in polyunsaturated fatty acids (PUFAs), and they are vulnerable to ROS that causes lipid peroxidation (LPO) [15].

Sperm and seminal plasma are endowed with enzymatic and nonenzymatic antioxidant compounds able to scavenge ROS [16]. However, semen laboratory processing such as centrifugation, cryopreservation, and, in general, semen handling leads to an increase in ROS production [17, 18]. A strategy to resolve this problem encompasses the supplementation of used media with antioxidants. With this purpose, many *in vitro* studies reported the scavenging ability of antioxidant compounds against oxidative stress induced to human sperm [19–23].

The aim of this study was to characterize the antioxidant potential of the extract of *Castanea sativa* Mill. (Bastarda Rossa) leaves and to evaluate its effect on oxidative stress induced upon human spermatozoa. The chemical analysis, performed by high-performance liquid chromatography-diode array detection (HPLC-DAD), enables the evaluation of the flavonoid and polyphenol content. The antiradical capacity was tested by the DPPH assay and electrochemical measurements. The effects of the *C. sativa* leaf extract were then assayed upon ejaculated human sperm in *in vitro* experiments. The possible toxicity of this extract was assessed by determining its effect at different dilutions on the sperm motility. The antioxidant ability of the extract against LPO [induced by hydrogen peroxide (H_2_O_2_)] was evaluated by measuring the levels of malondialdehyde (MDA) in human swim-up-selected sperm treated with H_2_O_2_ alone and H_2_O_2_ plus *C. sativa* extract. The morphological alterations induced by H_2_O_2_ upon spermatozoa and the protective effect of the extract were analyzed also by transmission electron microscopy (TEM).

## 2. Materials and Methods

### 2.1. *C. sativa* Extract Preparation

*Castanea sativa* Mill. var. Bastarda Rossa leaves were harvested in Arcidosso (Mount Amiata, Tuscany, Italy) in August, during their full development stage. Collection was performed in the morning, and leaves were manually chosen when they are intact and healthy and have no signs of microbial infection. Herbal material was dried to constant weight at 35°C.

The extraction procedure was accomplished using the automatic extractor Naviglio Estrattore® (Atlas Filtri, Padova, Italy) and validated and was very effective in obtaining an optimal yield of polyphenols from herbal drugs [24]. Briefly, dried *C. sativa* leaves were crushed and transferred into a 50 *μ*m extraction bag up to complete filling. The bag was placed in the extraction chamber that was filled with solvent of extraction (ethanol 75% *v*/*v*). Naviglio Estrattore® parameters were set as follows: static phase: 8 min/cycle, dynamic phase: 18 laps/cycle, and total cycles of extraction: 100. At the end of the extraction procedure, the extract was adjusted to a final drug : extract ratio 1 : 10.

### 2.2. Chemical Analysis

Total polyphenol and flavonoid content of the ethanolic extract (CEE) was examined using spectrophotometric methods reported in Biagi et al. [24]. In detail, total polyphenols were determined by the colorimetric method of Folin-Ciocalteu: 0.1 ml of each extract was added to 2.9 ml of distilled water and 0.5 ml of Folin-Ciocalteu reactive (1 : 10 *v*/*v*) to distilled water.

After 30 s of shaking, 1 ml of Na_2_CO_3_ 15% *w*/*w* was added to distilled water. After incubation at room temperature for 120 min, the absorbance at 700 nm was read using a SAFAS UV-MC2 instrument (Monte Carlo, Monaco). The polyphenol quantification was calculated by means of interpolation of the calibration curve constructed using gallic acid (Sigma-Aldrich, Milan, Italy).

Total flavonoid content of extracts was determined reading the absorbance at 353 nm of the extract diluted to 100-fold, according to Sosa et al. [25], and constructing a calibration curve using hyperoside as the standard (Sigma-Aldrich, Milan, Italy). All reagents were purchased from Sigma-Aldrich (Milan, Italy). A HPLC-DAD analysis was carried out to investigate the polyphenolic fraction of CEE and to identify the main chemical constituents. A Shimadzu Prominence LC 2030 3D instrument equipped with a Bondapak® C18 column (10 mm, 125 Å, 3.9 mm × 300 mm column) (Waters Corporation, USA) was used.

Water+0.1% *v*/*v* formic acid (A) and acetonitrile+0.1% *v*/*v* formic acid (B) were used as mobile phases. The following program was applied: B from 10% at 0 min to 25% at 20 min and then B 50% at 35 min; the flow was set at 0.8 ml/min. Chromatograms were recorded at 280 nm and 340 nm.

Analyses were performed using 10 *μ*l of CEE; hyperoside and quercetin (Sigma-Aldrich, Milan, Italy) were used as external standards. Calibration curves were established using reference standards ranging from 0.008 mg/ml to 0.5 mg/ml. The correlation coefficient (*R*^2^) of each curve was >0.99.

### 2.3. Antiradical Capacity: DPPH Assay

The antiradical capacity of CEE was tested by means of the validated DPPH (2,2-diphenyl-1-picrylhydrazyl) test. All reagents were purchased from Sigma-Aldrich, Milan, Italy. The DPPH solution was prepared in methanol at a concentration of 1 × 10^−4^ M. CEE was tested in seven 1 : 2 serial dilutions (ethanol 75% *v*/*v*), ascorbic acid, used as the reference, was dissolved in pure water, and hyperoside and quercetin were dissolved in ethanol 75% *v*/*v* and diluted in order to obtain a range of concentrations from 0.031 to 2 mg/ml. All the samples were mixed with the DPPH solution (1 : 19), transferred into 1 cm path length cuvettes, and incubated for 30 min at room temperature in the dark. Water or ethanol 75% *v*/*v* in DPPH (1 : 19) was used as the positive control. The inhibition of DPPH was calculated according to the following formula:

(1) $$\begin{array}{c} \%\text{inhibition}=\left(\frac{{\text{Abs}}_{\text{c}}-{\text{Abs}}_{\text{e}}}{{\text{Abs}}_{\text{c}}}\right)\times 100, \end{array}$$

where Abs_c_ is the absorbance of the positive control and Abs_e_ is the absorbance of the tested samples.

IC_50_ was calculated by constructing the curve of inhibition values for each tested concentration (in the linear range 10-75%) [22].

### 2.4. Electrochemical Measurements

Electrochemical experiments (cyclic voltammetry and differential pulse voltammetry (DPV)) were carried out to obtain information about redox properties of CEE as well as also of its main flavonoids such as hyperoside and quercetin. The electrochemical analyzer BAS100A (Bioanalytical Systems Inc., West Lafayette, USA) connected to a conventional three-electrode cell consisting of a glassy carbon working electrode, a platinum wire as an auxiliary electrode, and an Ag/AgCl reference electrode was used. The reported potentials were referred at a scan rate of 20 mV/s. Measures were acquired at room temperature under an ultrapure nitrogen inert atmosphere to prevent both the reactivity of the examined samples with the atmospheric oxygen and the dissolved oxygen reduction process. This analysis was carried out as follows:

- 1 ml of ethanolic solution of hyperoside 0.1 mg/ml was diluted in 5 ml of NaCl 0.1 M
- 1 ml of ethanolic solution of quercetin 0.1 mg/ml was diluted in 5 ml of NaCl 0.1 M
- 1 ml of ethanolic solution of CEE was diluted in 5 ml of NaCl 0.1 M

### 2.5. Semen Samples

Semen samples from 8 healthy men (aged 24-30 years) were collected by masturbation after 3–5 days of sexual abstinence. They were analyzed after liquefaction for 30 min at 37°C. Volume, pH, concentration, and motility were evaluated according to the World Health Organization guidelines [26]. All men provided an informed written consent before the inclusion in this study. The informed consent described the aims of the research and was approved by the Ethics Committee of Azienda Ospedaliera Universitaria Senese (CEAOUS).

### 2.6. Swim-Up to Select Motile Sperm

A swim-up technique was used to obtain the motile sperm fraction from 8 different ejaculates: 0.5 ml of each semen sample was placed in a sterile conical centrifuge tube and gently layered with 0.5 ml of Biggers-Whitten-Whittingham (BWW) medium [26]. The tubes, inclined at a 45° angle, were incubated for 45 min at 37°C and 5% CO_2_. Then, 0.5 ml of the uppermost medium that contains motile sperm fraction was collected and used for the experiments.

### 2.7. Determination of Sperm Motility after *C. sativa* Leaf Extract Treatment

Different dilutions of *C. sativa* leaf extracts in BWW medium (1 : 100, 1 : 200, and 1 : 500) were added to swim-up-selected sperm. Mixtures were incubated at 37°C and 5% CO_2_ for 1 h. Sperm motility was evaluated using a Burker counting chamber. Spermatozoa were categorized in different grades of motility (sperm with progressive and nonprogressive motility and immotile sperm) [26]. Aliquots of the selected sperm treated in the same conditions but without the *C. sativa* leaf extract were used as controls. All experiments were carried out eight times, and results were reported as mean values ± standard deviation (SD) and median.

### 2.8. LPO Induction and *C. sativa* Leaf Extract Treatment

Each sample of swim-up-selected human sperm was separated into aliquots, which were composed of sperm alone, sperm treated with 100 *μ*M H_2_O_2_ and the leaf extract diluted to 1 : 100, 1 : 200, and 1 : 500 in BWW medium, and sperm treated with 100 *μ*M H_2_O_2_ alone. Aliquots were incubated at 37°C and 5% CO_2_ for 1 h. After incubation, the aliquots were centrifuged at 200 g to separate BWW and spermatozoa. The supernatant was stored at -80°C until levels of MDA were assessed.

### 2.9. Malondialdehyde (MDA) Level Assessment

The extent of LPO was estimated assessing free MDA concentrations according to Shara et al. [27]. After thawing, 500 *μ*l of each sample was added to 500 *μ*l of 0.04 M Tris(hydroxymethyl) methylamine (pH 7.4) and 0.01% butyl hydroxytoluene in acetonitrile (1 : 1 *v*/*v*) to avoid the artificial oxidation of polyunsaturated free fatty acids during the assay. The samples were centrifuged at 3000 g at 4°C for 15 min. The supernatant was used for MDA analysis after precolumn derivatization with 2,4-dinitrophenylhydrazine. The MDA-hydrazone was quantified by isocratic reversed-phase HPLC (Waters 600 E System Controller HPLC equipped with a Waters Dual *λ* 2487 detector, Milford, MA, USA) with UV detection at 307 nm. MDA values were reported as nmol/ml.

### 2.10. Transmission Electron Microscopy (TEM)

Human sperm treated with 100 *μ*M H_2_O_2_ and the *C. sativa* leaf extract diluted to 1 : 200 in BWW and with 100 *μ*M H_2_O_2_ alone were processed for TEM. Sperm samples were fixed in cold Karnovsky fixative at 4°C for 2 h; then, they were washed in 0.1 mol/l cacodylate buffer (pH 7.2) for 12 h, postfixed in 1% buffered osmium tetroxide for 1 h at 4°C, dehydrated, and embedded in Epon Araldite. Ultrathin sections were cut with a Supernova Ultramicrotome (Reichert Jung, Vienna, Austria), mounted on copper grids, stained with uranyl acetate and lead citrate, and observed and photographed with a Philips CM12 TEM (Philips Scientific, Eindhoven, the Netherlands; Centro di Microscopie Elettroniche “Laura Bonzi,” ICCOM, Consiglio Nazionale delle Ricerche (CNR), Via Madonna del Piano, 10, Firenze, Italy).

At least 300 sperm sections were analyzed for each sample, and the anomalies related to the acrosome, the chromatin, the axoneme, and the plasma membrane were quantified. These experiments were performed three times.

### 2.11. Statistical Analysis

The statistical elaboration was performed with software R for statistical computing, open source for Windows, version 3.3.1 [R Core Team (2016). R: A language and environment for statistical computing. R Foundation for Statistical Computing, Vienna, Austria. URL https://www.R-project.org/]. The comparisons of sperm motility percentages and MDA levels in samples treated with 100 *μ*M H_2_O_2_ and *C. sativa* leaf extracts at different dilutions were calculated by the Kruskal–Wallis test. When a statistically significant difference was found among the groups, the Wilcoxon rank sum test was then used between pairs of groups. The comparisons between the percentages of sperm organelle alterations after incubation with H_2_O_2_ and the *C. sativa* leaf extract diluted to 1 : 200 were calculated by the Kruskal–Wallis test. *P* < 0.05 was considered significant.

## 3. Results

### 3.1. Chemical Analysis of *C. sativa* leaf extract

The Folin-Ciocalteu colorimetric assay and direct spectrophotometry enabled the quantification of the total polyphenols and total flavonoids in CEE, respectively.  Table 1 shows the quantifications of total polyphenols expressed as gallic acid equivalents (mg/l of extract) and total flavonoids expressed as hyperoside (mg/l of extract). Results showed that about one-third of the polyphenols in the extract are flavonoid type; it was plausible to refer the remaining polyphenolic portion to phenolic acids, hydroxycinnamic derivatives, and tannins.

**Figure 1:**
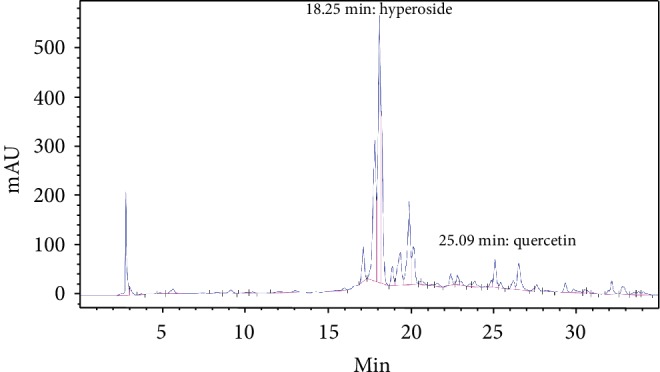
DAD spectrum of CEE recorded at 340 nm. The main CEE monomeric polyphenols were represented by quercetin glycosides, such as hyperoside (RT = 18.25 min). Quercetin is also present in the extract, but in low concentration (RT = 25.09 min).

HPLC-DAD analysis recorded 5 main constituents in CEE at the following retention times (RT): 17.85 min, 18.13 min, 18.25 min, 19.31 min, and 19.91 min (Figure 1). With the exception of the constituent at RT 17.85 min, all the other major constituents were found to be flavonoids, showing a characteristic UV profile of this class of metabolites.

From the comparison with the standards and the literature, it was possible to assign the peak at 18.25 min to the hyperoside (Figure 1), while the peak at 18.13 min is with high probability an isomer of the hyperoside, plausibly isoquercitrin. Quercetin is also present in the extract, but in low concentration (RT = 25.09 min, 6.30 mg/l). The main nonflavonoid constituent can be assigned to the catechin tannin class, but it was not identifiable not being referable to the used reference standards. In CEE, the concentration of hyperoside was 75.28 mg/l.

### 3.2. Antiradical Capacity: DPPH Assay

The DPPH assay was carried out on CEE to measure its antiradical capacity. Free radical inhibition values were linearly correlated with CEE concentration in the concentration range 0.05%-0.2%, while at higher concentrations, the typical maximal inhibition plateau was obtained.

From the construction of the correlation line between concentration and activity in the linear zone, it was possible to calculate the IC_50_ (inhibitor concentration that reduces the activity by 50%) of CEE, which was found to be 0.081% (0.72 mg/ml). Results were validated using ascorbic acid as the reference IC_50_ < 10 *μ*g/ml [28]. The CEE phytocomplex exerted a synergistic effect compared to the main single compounds. Indeed, repeating the DPPH test on hyperoside and quercetin, we found that the best IC_50_ value was exerted by quercetin 0.01 mg/ml, while the IC_50_ value of hyperoside was 0.06 mg/ml; these values were higher than those which one would expect considering the presence of these compounds at the IC_50_ value of CEE (when CEE diluted at 0.081% contains 0.06 mg/l of hyperoside and 0.005 mg/l of quercetin).

### 3.3. Electrochemical Measurements

**Figure 2:**
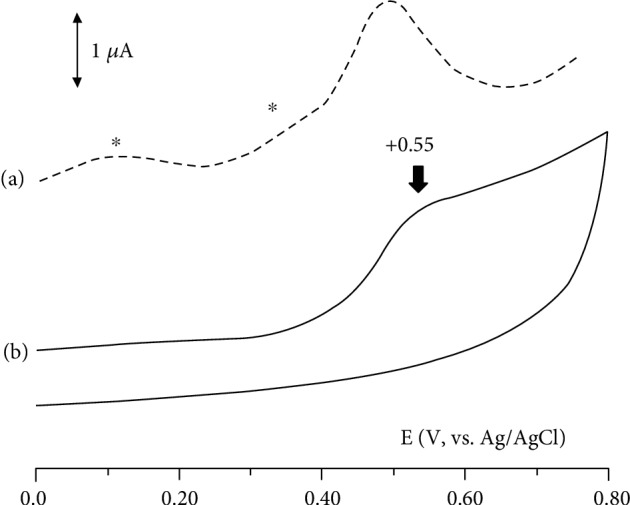
Differential pulse voltammogram (DPV) (a) and cyclic voltammogram (b) recorded for CEE 1 : 5 in water solution on a glassy carbon electrode. Scan rate: 20 mV/s. An anodic process (oxidation) at +0.55 V is recorded by differential pulse voltammetry and cyclic voltammetry methods. Other oxidation peaks were detected in DPV (asterisks).

Cyclic voltammograms recorded in the solution containing quercetin showed a quasireversible oxidation process with an anodic peak at +0.33 V. The same profile was recorded in the solution containing hyperoside, but the anodic peak potential was reached at +0.44 V (data not shown in figures). More complicated redox behaviour was recorded in the extract solution. Indeed, the cyclic voltammogram of the CEE solution (Figure 2, curve b arrow) showed a main irreversible oxidation process at +0.55 V; however, the differential pulse voltammetry, analysis more sensitive than cyclic voltammetry, highlighted that CEE undergoes two other cathodic processes with peak potentials around +0.15 V and +0.40 V (Figure 2, curve a asterisks). It is interesting to note that both peak values are lower than that measured in hyperoside solution (+0.44 V) supporting the highest antioxidant activity recorded for CEE.

### 3.4. Effect of the *C. sativa* Leaf Extract on Sperm Motility

The effect of the *C. sativa* leaf extract on motility of swim-up-selected sperm was assessed. The seminal parameters of the used samples ranged from the 10th to 50th centile reported in the WHO guidelines [26]. Results showed that the *C. sativa* leaf extract used at the dilutions of 1 : 100, 1 : 200, and 1 : 500 did not have an effect on sperm motility (Table 2).

### 3.5. Effect of the *C. sativa* Leaf Extract on Induced LPO in Sperm Samples: MDA Evaluation

**Figure 3:**
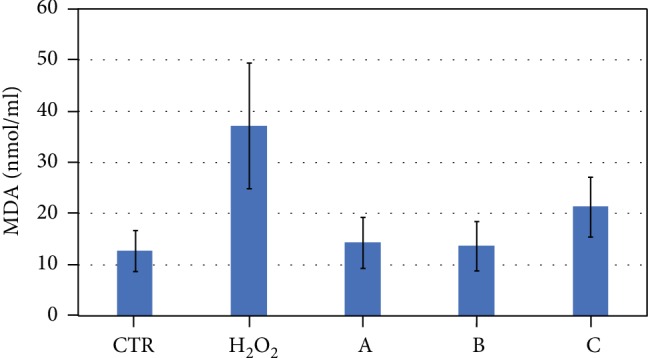
Mean values and standard error of MDA levels measured in swim-up-selected samples treated as follows: controls (CTR), aliquots treated with H_2_O_2_, and specimens treated with H_2_O_2_+*C. sativa* leaf extract 1 : 100 (A), H_2_O_2_+*C. sativa* leaf extract 1 : 200 (B), and H_2_O_2_+*C. sativa* leaf extract 1 : 500 (C). Kruskal–Wallis test: *P* = 0.02913. H_2_O_2_ vs. CTR: *P* = 0.006993. H_2_O_2_ vs. A: *P* = 0.01476. H_2_O_2_ vs. B: *P* = 0.01571.

Swim-up-selected sperm were treated with H_2_O_2_ to induce LPO and with both H_2_O_2_ and *C. sativa* leaf extract diluted to 1 : 100, 1 : 200, and 1 : 500 in order to test the potential scavenging activity of this compound. MDA levels were measured in the supernatant, and the results are shown in Figure 3. The level of MDA significantly increases after H_2_O_2_ treatment (37.21 ± 12.24 nmol/ml) with respect to control (12.73 ± 3.98 nmol/ml, *P* = 0.006993). The MDA levels in samples incubated with H_2_O_2_ plus *C. sativa* leaf extract diluted to 1 : 100 (14.33 ± 5.00 nmol/ml, *P* = 0.01476) and 1 : 200 (13.65 ± 4.81, *P* = 0.01571) were significantly lower than those measured in the sample treated with H_2_O_2_ alone and were similar to the levels measured in the control specimens. No significant difference was found between MDA levels in sperm treated with both H_2_O_2_ and *C. sativa* leaf extract diluted to 1 : 500 (21.33 ± 5.81 nmol/ml) and those in sperm treated with H_2_O_2_ alone.

### 3.6. Effect of the *C. sativa* Leaf Extract on Induced LPO in Sperm Samples: Ultramorphological Evaluation

**Figure 4:**
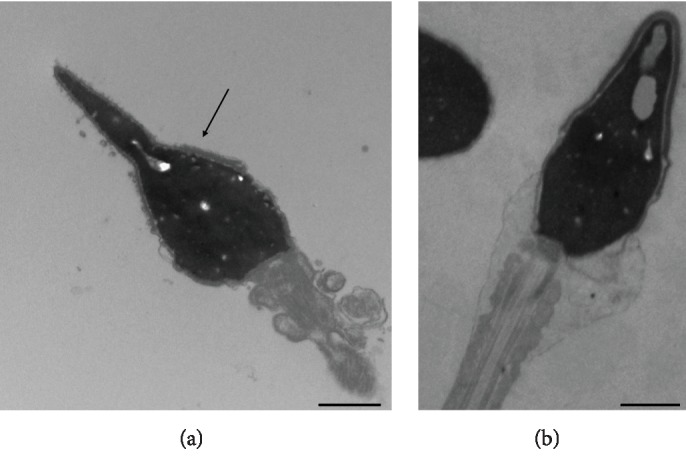
TEM micrographs of human sperm treated with H_2_O_2_ without the *C. sativa* leaf extract (a) and H_2_O_2_ with the *C. sativa* leaf extract diluted to 1 : 200 (b). In (a), a spermatozoon with the reacted acrosome (arrow) and altered chromatin texture, particularly evident along the edge of the cell, is shown. The membrane is broken. In (b), a normal spermatozoon with integer structures is shown. Bars: 1 *μ*M.

**Figure 5:**
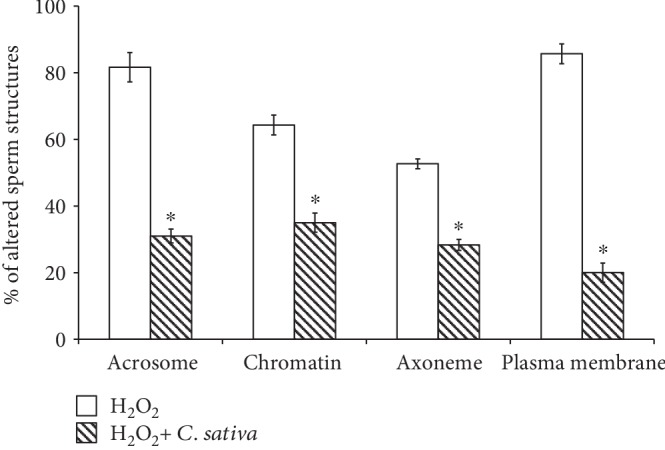
Mean values and standard errors of percentages of altered sperm structures analyzed by TEM in samples treated as follows: aliquots treated with H_2_O_2_ and specimens treated with H_2_O_2_ and *C. sativa* leaf extract 1 : 200. H_2_O_2_ vs. H_2_O_2_+*C. sativa*: *P* < 0.05.

For TEM experiments, we used the dilution of 1 : 200 as it represented the lowest concentration that exhibited the highest antioxidant power out of the tested dilutions. The damage observed in sperm incubated with H_2_O_2_ consisted of broken plasma membranes, reacted and absent acrosomes, chromatin with altered texture (Figure 4(a)), and flagella with altered periaxonemal and axonemal cytoskeletal elements. The *C. sativa* leaf extract diluted to 1 : 200 showed a protective activity towards the considered alterations induced by LPO (Figure 4(b)). In particular, the percentages of sperm with a broken plasma membrane (85.67 ± 2.96 vs. 20 ± 2.89, *P* < 0.05), with the absent acrosome (81.66 ± 4.41 vs. 31.66 ± 2.08, *P* < 0.05), with altered chromatin (64.33 ± 2.96 vs. 35 ± 2.88, *P* < 0.05), and with anomalies in axonemal and periaxonemal structures (52.67 ± 1.45 vs. 28.33 ± 1.67, *P* < 0.05) were significantly reduced in samples treated with the *C. sativa* leaf extract (Figure 5).

## 4. Discussion

In this research, we characterized a 75% *v*/*v* ethanolic leaf extract of a typical Tuscan chestnut variety: “Bastarda Rossa” from Mount Amiata. *C. sativa* leaves are the least exploited by-product of this species, despite the fact that they are rich in phenolic compounds, which are endowed with antibacterial and DNA-protective activities and are efficient in the prevention and treatment of oxidative stress-mediated diseases such as photoaging [29].

A 70% *v*/*v* ethanolic extract of *C. sativa* leaves from Mirandela (Portugal) was investigated and resulted to be very effective in *in vitro* scavenging and antioxidant tests. In a cutaneous irritation test, performed in 20 healthy people, it resulted to be well tolerated and free from side effects [30]. Barreira et al. [31], in a comparative study, reported that antioxidant potential of chestnut leaves was higher than that of almond leaf extracts.

Chemical analysis conducted in this work showed that CEE contains a concentration of total polyphenols (3475.23 mg/l) higher than that of other well-studied antioxidant natural products such as red wine [32] and green and black teas from *Camellia sinensis* Kuntze leaves [33]. Interestingly, a large part of these polyphenols is represented by monomeric flavonoids; in particular, hyperoside and quercetin glycosides were the most abundant constituents. Recently, great attention has been focused on hyperoside for its effective antioxidant capacity in an innovative model of oxidative stress using the yeast *Saccharomyces cerevisiae* [34]. Hyperoside also showed cytoprotective action against H_2_O_2_-induced cell damage in Chinese hamster lung fibroblast cells, exerting intracellular scavenging activity [35]. In addition, quercetin and its main glycosides, such as rutin and isoquercitrin, are well-known antioxidant molecules [36].

By monitoring the qualitative profile of the recorded chromatogram and according to Cerulli et al. [12] who studied the methanol extract of the leaves of *C. sativa* var. “Marrone di Roccadaspide,” it is plausible to refer that phenolic acids, hydroxycinnamic derivatives, and tannins are also present in the CEE. To determine the antioxidant capacity of the chestnut leaf extract, we employed either the classic and validated DPPH test or the cyclic voltammetry, an interesting, but least exploited, technique. CEE exerted a good antiradicalic capacity in the DPPH test, showing to be effective even diluted to over 1000-fold. The IC_50_ value of the extract is comparable with that of a polyphenol-enriched red wine [37]. In spite of its simplicity, the qualitative conventional electroanalysis has a prominent role in the assessment of antioxidants in food and biological samples and in the evaluation of their antioxidant activity *in vitro.* The relationship between the sample voltammogram and its *in vitro* antioxidant activity was defined by its oxidation potential: peaks with low values of oxidation potential were associated with high antioxidant activity while peaks with the high values were associated with low antioxidant capacities. The high sensitivity of DPV enables pointing out processes with low peak current, which is difficult to highlight with cyclic voltammetry. In our extract, these peaks were present at peak potential values of +0.15 to +0.40 V and +0.55 V, with the last one evident also in the cyclic voltammogram. All these potential peaks are associated with a good antioxidant activity according to the electrochemical behaviour typical of flavonoids [38].

In particular, the peaks located at more cathodic potential values than quercetin and hyperoside indicate that the chemical composition of the extract includes minor components which are responsible for the antioxidant activity of CEE.

These findings related to the redox characteristics of the chestnut extract implemented those obtained in the evaluation of antiradicalic capacity with DPPH and highlighted the antioxidant characteristics of the extract phytocomplex.

The experimental protocol performed in this work confirmed that the chestnut leaf extract of Bastarda Rossa is worthy to be investigated for its interesting biological characteristics. For this reason, we decided to explore the ability of the *C. sativa* leaf extract to prevent LPO induced *in vitro* by H_2_O_2_ on swim-up-selected human sperm. Our final purpose was to find out natural substances that could be used as antioxidant supplementations in fertilization culture media.

It is known that semen processing for assisted reproduction technologies and cryopreservation requires protocols that include centrifugation and incubation of spermatozoa in culture media and that these procedures could exacerbate oxidative stress and induce damage to sperm cells [39] due to ROS generation. Spermatozoa, particularly human ones, are vulnerable to ROS attack. ROS may cause LPO of sperm membranes given that they are particularly rich in unsaturated fatty acids. The LPO process leads to a loss of membrane integrity, an increase in its permeability, inactivation of cellular enzymes, DNA damage, and cell apoptosis. This problem is not only related to semen handling, since it is estimated that semen samples from 25% *ca.* of infertile men present elevated levels of ROS and, often, decreased antioxidant capacity [40]. The oral supplementation of antioxidant substances may improve semen parameters and oxidant/antioxidant status in infertile males [41]. Therefore, it seems reasonable to support the treatment of male infertility with substances able to neutralize ROS. In this particular field, the study of natural compounds endowed with antioxidant properties is appealing, either from the point of view of male infertility treatment or for the development of new strategies for the supplementation *in vitro* of media used for semen handling.

We have previously demonstrated the scavenging activity of some natural compounds, such as resveratrol, quercetin, rutin, naringenin, epicatechin, and Propolfenol®, a phytocomplex rich in European propolis and catechins, against LPO induced *in vitro* to human spermatozoa [20–22, 42]. Recently, Kedechi et al. [39] demonstrated the antioxidant effect of hydroxytyrosol, the main phenolic compound of virgin oil, during sperm centrifugation. In addition, in a mouse model, it was found that the supplementation of media with antioxidants during all stages of the *in vitro* fertilization procedures and cultures could play a beneficial effect on assisted reproduction and may preserve embryo viability [43].

The results of this study demonstrated that the *C. sativa* leaf extract used at dilutions of 1 : 100, 1 : 200, and 1 : 500 did not show any toxicity on sperm motility. These results are different from those obtained by some of our group using purified polyphenols [20, 21], which, at high concentration, decreased sperm motility and viability and damaged DNA, as we observed using liposomes loaded with 100 *μ*M quercetin [42].

It seems that a combination of natural antioxidant compounds is more tolerated than single purified compounds and that the phytocomplex exerts a synergistic action, at least in this kind of *in vitro* experiments, as it was observed also when we used Propolfenol® in similar protocols [22]. Actually, the multitarget features and aspecific mechanisms are the rationale for the use of phytocomplexes in modern phytotherapy. The *C. sativa* leaf extract, at dilutions of 1 : 100 and 1 : 200, showed a scavenging activity on H_2_O_2_-induced LPO, restoring similar levels of MDA measured in the control samples. To visualize the protective activity of the extract, a deep analysis of the single sperm organelles was performed using TEM that is a useful method to explore the morphological variation of human sperm treated *in vitro* with different compounds [20, 21]. In general, TEM is one of the methods to study the different organelles characterizing sperm defects that can influence the fertilizing potential [44, 45]. In this research, we demonstrated that the *C. sativa* leaf extract diluted to 1 : 200 was powerful in protecting sperm against severe oxidative damage induced by H_2_O_2_. The extract was able to defend sperm membranes and the acrosomes, both structures particularly affected by H_2_O_2_ treatment.

## 5. Conclusions

The leaf extract of *Castanea sativa* Mill. var. Bastarda Rossa (Mount Amiata, Italy) is particularly rich in polyphenols and flavonoids and shows scavenging properties against oxidative stress induced by H_2_O_2_ to human ejaculated sperm. This extract could be used in protocols of gamete handling such as techniques of assisted reproduction and cryopreservation of semen, all conditions in which oxidative stress is exacerbated. We are aware that further studies are needed to confirm the antioxidant ability of the *C. sativa* leaf extract. Should such activity be corroborated, these studies could be extended to the field of farming animals, in which the cryopreservation procedures are particularly common.

## Figures and Tables

**Table 1:** The quantification of total polyphenols expressed as gallic acid and total flavonoids expressed as hyperoside (mg/l of extract).

Sample	Total polyphenols expressed as gallic acid (mg/l)	Total flavonoids expressed as hyperoside (mg/l)
CEE	3475.23 ± 144.05	1081.83 ± 66.55

**Table 2:** Mean ± standard deviation and median (values in italics) of progressive sperm motility % evaluated in controls and samples treated with 1 : 100, 1 : 200, and 1: 500 *C. sativa* leaf extracts.

Sperm parameter	Control	*C. sativa* 1 : 100	*C. sativa* 1 : 200	*C. sativa* 1 : 500	Kruskal–Wallis test
Progressive sperm motility %	68.37 ± 10.97 *69*	68.5 ± 13.41 *69*	68 ± 11.93 *68*	68.25 ± 11.41 *73*	*P* = 0.9731

## Data Availability

The data used to support the findings of this study are included within the article.

## References

[1] Braga N., Rodrigues F., Oliveira M. B. (2015). Castanea sativa by-products: a review on added value and sustainable application. *Natural Product Research*.

[2] Chiarini A., Micucci M., Malaguti M. (2013). Sweet chestnut (*Castanea sativa* Mill.) bark extract: cardiovascular activity and myocyte protection against oxidative damage. *Oxidative Medicine and Cellular Longevity*.

[3] Basile A., Sorbo S., Giordano S. (2000). Antibacterial and allelopathic activity of extract from *Castanea sativa* leaves. *Fitoterapia*.

[4] Santulli C., Brizi C., Micucci M. (2017). *Castanea sativa* Mill. bark extract protects U-373 MG cells and rat brain slices against ischemia and reperfusion injury. *Journal of Cellular Biochemistry*.

[5] Budriesi R., Vivarelli F., Canistro D. (2018). Liver and intestinal protective effects of *Castanea sativa* Mill. bark extract in high-fat diet rats. *PLoS One*.

[6] Dìaz Reinoso B., Couto D., Moure A., Fernandes E., Domìnguez H., Parajò J. C. (2012). Optimization of antioxidants – extraction from *Castanea sativa* leaves. *Chemical Engineering Journal*.

[7] Almeida I. F., Costa P. C., Bahia M. F. (2010). Evaluation of functional stability and batch-to-batch reproducibility of a *Castanea sativa* leaf extract with antioxidant activity. *AAPS PharmSciTech*.

[8] Craft B. D., Kerrihard A. L., Amarowicz R., Pegg R. B. (2012). Phenol-based antioxidants and the *in vitro* methods used for their assessment. *Comprehensive Reviews in Food Science and Food Safety*.

[9] Gomes A., Fernandes E., Silva A. M. S. (2007). 2-Styrylchromones: novel strong scavengers of reactive oxygen and nitrogen species. *Bioorganic & Medicinal Chemistry*.

[10] Mujić A., Grdović N., Mujić I. (2011). Antioxidative effects of phenolic extracts from chestnut leaves, catkins and spiny burs in streptozotocin-treated rat pancreatic *β*-cells. *Food Chemistry*.

[11] Quave C. L., Lyles J. T., Kavanaugh J. S. (2015). *Castanea sativa* (European chestnut) leaf extracts rich in ursene and oleanene derivatives block *Staphylococcus aureus* virulence and pathogenesis without detectable resistance. *PLoS One*.

[12] Cerulli A., Masullo M., Mari A. (2018). Phenolics from *Castanea sativa* leaves and their effects on UVB-induced damage. *Natural Product Research*.

[13] Dutta S., Majzoub A., Agarwal A. (2019). Oxidative stress and sperm function: a systematic review on evaluation and management. *Arab Journal of Urology*.

[14] Zini A., Al-Hathal N. (2011). Antioxidant therapy in male infertility: fact or fiction?. *Asian Journal of Andrology*.

[15] Sikka S. C. (2001). Relative impact of oxidative stress on male reproductive function. *Current Medicinal Chemistry*.

[16] Ko E. Y., Sabanegh E. S., Agarwal A. (2014). Male infertility testing: reactive oxygen species and antioxidant capacity. *Fertility and Sterility*.

[17] Agarwal A., Ikemoto I., Loughlin K. R. (1994). Effect of sperm washing on levels of reactive oxygen species in semen. *Archives of Andrology*.

[18] Aitken R. J., Finnie J. M., Muscio L. (2014). Potential importance of transition metals in the induction of DNA damage by sperm preparation media. *Human Reproduction*.

[19] Ben Abdallah F., Zribi N., Ammar-Keskes L. (2011). Antioxidative potential of quercetin against hydrogen peroxide induced oxidative stress in spermatozoa *in vitro*. *Andrologia*.

[20] Collodel G., Federico M. G., Geminiani M. (2011). Effect of trans-resveratrol on induced oxidative stress in human sperm and in rat germinal cells. *Reproductive Toxicology*.

[21] Moretti E., Mazzi L., Terzuoli G. (2012). Effect of quercetin, rutin, naringenin and epicatechin on lipid peroxidation induced in human sperm. *Reproductive Toxicology*.

[22] Biagi M., Collodel G., Corsini M., Pascarelli N. A., Moretti E. (2018). Protective effect of Propolfenol^®^ on induced oxidative stress in human spermatozoa. *Andrologia*.

[23] Alamo A., Condorelli R. A., Mongioì L. M. (2019). Environment and male fertility: effects of benzo-*α*-pyrene and resveratrol on human sperm function in vitro. *Journal of Clinical Medicine*.

[24] Biagi M., Manca D., Barlozzini B., Miraldi E., Giachetti D. (2014). Optimization of extraction of drugs containing polyphenols using an innovative technic. *Agro Food Industry Hi-Tech*.

[25] Sosa S., Bornancin A., Tubaro A., Loggia R. D. (2007). Topical antiinflammatory activity of an innovative aqueous formulation of actichelated propolis vs two commercial propolis formulations. *Phytotherapy Research*.

[26] World Health Organization (2010). *WHO Laboratory Manual for the Examination and Processing of Human Semen*.

[27] Shara M. A., Dickson P. H., Bagchi D., Stohs S. J. (1992). Excretion of formaldehyde, malondialdehyde, acetaldehyde and acetone in the urine of rats in response to 2,3,7,8-tetrachlorodibenzo-*p*-dioxin, paraquat, endrin and carbon tetrachloride. *Journal of Chromatography B: Biomedical Sciences and Applications*.

[28] Nariya P. B., Nariya M. B., Shukla V. J., Acharya R., Bhalodia N. R. (2013). *In vitro* evaluation of antioxidant activity of *Cordia dichotoma* (Forst f.) bark. *AYU*.

[29] de Vasconcelos M. C., Bennett R. N., Rosa E. A., Ferreira-Cardoso J. V. (2010). Composition of European chestnut (*Castanea sativa* Mill.) and association with health effects: fresh and processed products. *Journal of the Science of Food and Agriculture*.

[30] Almeida I. F., Valentão P., Andrade P. B. (2008). In vivo skin irritation potential of a *Castanea sativa* (chestnut) leaf extract, a putative natural antioxidant for topical application. *Basic & Clinical Pharmacology & Toxicology*.

[31] Barreira J. C., Ferreira I. C., Oliveira M. B., Pereira J. A. (2010). Antioxidant potential of chestnut (Castanea sativa L.) and almond (Prunus dulcis L.) by-products. *Food Science and Technology International*.

[32] Giovinazzo G., Carluccio M. A., Grieco F., Mérillon J. M., Ramawat K. (2019). Wine polyphenols and health. *Bioactive Molecules in Food*.

[33] Gulua L., Nikolaishvili L., Jgenti M., Turmanidze T., Dzneladze G. (2018). Polyphenol content, anti-lipase and antioxidant activity of teas made in Georgia. *Annals of Agrarian Science*.

[34] Gao Y., Fang L., Wang X. (2019). Antioxidant activity evaluation of dietary flavonoid hyperoside using saccharomyces cerevisiae as a model. *Molecules*.

[35] Piao M. J., Kang K. A., Zhang R. (2008). Hyperoside prevents oxidative damage induced by hydrogen peroxide in lung fibroblast cells via an antioxidant effect. *Biochimica et Biophysica Acta (BBA) - General Subjects*.

[36] Williamson G., Plumb G. W., Uda Y., Price K. R., Rhodes M. J. (1996). Dietary quercetin glycosides: antioxidant activity and induction of the anticarcinogenic phase II marker enzyme quinone reductase in Hepalclc7 cells. *Carcinogenesis*.

[37] Leahu A., Amariei S., Damian C., Oroian M., Ropciuc S. (2014). Evaluation of the antioxidant activity of some types of red and white wines. *Ovidius University Annals of Chemistry*.

[38] Gil E. S., Couto R. O. (2013). Flavonoid electrochemistry: a review on the electroanalytical applications. *Revista Brasileira de Farmacognosia*.

[39] Kedechi S., Zribi N., Louati N. (2017). Antioxidant effect of hydroxytyrosol on human sperm quality duringin vitroincubation. *Andrologia*.

[40] Walczak-Jedrzejowska R., Wolski J. K., Slowikowska-Hilczer J. (2013). The role of oxidative stress and antioxidants in male fertility. *Central European Journal of Urology*.

[41] Jannatifar R., Parivar K., Roodbari N. H., Nasr-Esfahani M. H. (2019). Effects of N-acetyl-cysteine supplementation on sperm quality, chromatin integrity and level of oxidative stress in infertile men. *Reproductive Biology and Endocrinology*.

[42] Moretti E., Mazzi L., Bonechi C. (2016). Effect of quercetin-loaded liposomes on induced oxidative stress in human spermatozoa. *Reproductive Toxicology*.

[43] Truong T., Gardner D. K. (2017). Antioxidants improve IVF outcome and subsequent embryo development in the mouse. *Human Reproduction*.

[44] Moretti E., Sutera G., Collodel G. (2016). The importance of transmission electron microscopy analysis of spermatozoa: diagnostic applications and basic research. *Systems Biology in Reproductive Medicine*.

[45] Chemes H. E. (2018). Phenotypic varieties of sperm pathology: genetic abnormalities or environmental influences can result in different patterns of abnormal spermatozoa. *Animal Reproduction Science*.

